# Embedding resilience in the design of the electricity supply for industrial clients

**DOI:** 10.1371/journal.pone.0188875

**Published:** 2017-11-30

**Authors:** Márcio das Chagas Moura, Helder Henrique Lima Diniz, Enrique López Droguett, Beatriz Sales da Cunha, Isis Didier Lins, Vicente Ribeiro Simoni

**Affiliations:** 1 Center for Risk Analysis and Environmental Modeling – CEERMA, Universidade Federal de Pernambuco, Recife, Pernambuco, Brazil; 2 Department of Production Engineering, Universidade Federal de Pernambuco, Recife, Pernambuco, Brazil; 3 Mechanical Engineering Department, University of Chile, Santiago, Chile; 4 Mechanical Engineering Department, University of Maryland, College Park, Maryland, United States of America; 5 Companhia Hidro Elétrica do São Francisco – Chesf, Recife, Pernambuco, Brazil; US Army Engineer Research and Development Center, UNITED STATES

## Abstract

This paper proposes an optimization model, using Mixed-Integer Linear Programming (MILP), to support decisions related to making investments in the design of power grids serving industrial clients that experience interruptions to their energy supply due to disruptive events. In this approach, by considering the probabilities of the occurrence of a set of such disruptive events, the model is used to minimize the overall expected cost by determining an optimal strategy involving pre- and post-event actions. The pre-event actions, which are considered during the design phase, evaluate the resilience capacity (absorption, adaptation and restoration) and are tailored to the context of industrial clients dependent on a power grid. Four cases are analysed to explore the results of different probabilities of the occurrence of disruptions. Moreover, two scenarios, in which the probability of occurrence is lowest but the consequences are most serious, are selected to illustrate the model’s applicability. The results indicate that investments in pre-event actions, if implemented, can enhance the resilience of power grids serving industrial clients because the impacts of disruptions either are experienced only for a short time period or are completely avoided.

## Introduction

Systems such as those for the distribution of electricity, water, oil, material supplies, and electronic communications correspond to Critical Infrastructures (CIs) by providing fundamental services to the economy and the routine operation of society. Many elements of CIs take the form of networks [1], with dependency among nodes and links, which in turn are usually interconnected with other networks. The efficiency of an entire CI depends on the availability of each element [2]; therefore, the occurrence of undesired and unexpected events, such as natural disasters, bad weather or a combination of other factors, can cause adverse and extended effects on the system, leading to social, environmental and economic impacts, although the probability of such events is usually low [3,4].

In this context, Electric Power Supply Networks (EPSNs) are especially critical because other CIs rely on electricity to manage and operate their processes [5]. Data from several studies estimate that the annual costs to the U.S. economy due to blackouts are between US$ 20 billion and US$ 55 billion [6]. The impact of power outages on the manufacturing industry involves losses of output volume and quality, inventory and asset damage, and production delays and inconveniences [7].

A survey conducted by the Brazilian National Confederation of Industry [8] showed that electrical energy is the primary power source of nearly 80% of factories located in Brazil, of which 67% stated that power supply interruptions significantly increase production costs. For instance, in 2012, a set of factories in Midwest Brazil suffered a total loss of US$ 2 million due to disruptions in the power supply [9]. In this context, EPSNs and their industrial customers must be resilient and sufficiently flexible to overcome the consequences of the occurrence of disruptive events as rapidly and economically as possible.

Although the resilience concept has become increasingly important, there remain a significant number of distinct definitions, demonstrating a lack of standardization to evaluate resilience, both qualitatively and quantitatively [5,10–14]. This paper understands the concept of resilience as the ability of the system to reduce both the magnitude and the duration of deviations from target performance levels, given the occurrence of undesired events [10,15–17].

According to Turnquist and Vugrin [1], models that focus on post-event strategies can frequently be time consuming, and they do not guarantee that one will identify an optimal or near-optimal set of actions that enable the most effective recovery for a variety of potential disruption scenarios. Thus, it is expected that pre-event strategies tend to be more efficient, useful, and profitable, especially when implemented during the design phase of a system. According to Linkov [10], strategies to build resilience during a system’s design phase can either minimize performance loss or increase recovery speed through redundancy, modularity, flexibility and independency between elements.

Despite this finding, most of the research on resilience has focused on post-event policies, as seen in [3,11–16], and the design of resilient systems remains a topic with limited research [16]. However, there has been a trend for decision makers to change from a reactive stance to a proactive one; consequently, the concept of resilience has been increasingly incorporated into systems’ design phases [17]. Moreover, a limited number of quantitative works focusing both on resilience and on the variables that affect system performance, such as cost of operation, customer service and investments in design [17,18]. Therefore, designing a resilient power grid is a prominent area for study because of its potential to enable improvements in network performance and thus to provide benefits to customers by enhancing the service level, regularity and quality of the power supply.

Therefore, the main objective of this paper is to develop a quantitative model that determines the optimal allocation of financial resources to establish a resilience-based strategy. To this end, we consider the expected financial impacts of uncertain disruptive scenarios and confront them with a set of strategies of investment to support decisions related to enhancing power grid resilience. Thus, similar to [1], our problem is modelled using Mixed-Integer Linear Programming (MILP) with the overall expected costs as the objective function, including the costs of pre-event decisions, the expected costs arising from the financial impact of disruptive scenarios on the network and the expected costs of post-event actions.

We present an application example to illustrate the applicability of the proposed model. Four cases are analysed to explore the results for different situations regarding the probability of the occurrence of disruptive scenarios. The resilience-based strategy defined for each case minimizes the total expected costs and is analysed in terms of power grid overall performance, involving power grid configuration, demand satisfied and recovery time. Moreover, two individual scenarios are analysed, demonstrating how the model can be applied to propose an appropriate resilience-based strategy for a specific situation.

Sensitivity analysis is also conducted to evaluate the impact of financial constraints for design investments compared to the overall performance of the power grid and the overall cost. The results demonstrate that higher investments during the design phase, when optimally allocated, have the potential to improve power grid performance and still reduce overall costs.

The remainder of the paper is organized as follows. Section 2 presents the theoretical background on resilience, including different approaches, applications and comparisons with other concepts. Section 2 also introduces useful concepts about EPSNs and their state of the art in the context of resilience. Section 3 shows the characteristics of the EPSN considered and the formulation of the proposed optimization model. Section 4 discusses examples to illustrate the applicability of the model. Finally, Section 5 concludes with remarks.

## Theoretical background

### The concept of resilience

Resilience assessment requires information about the disruptive events which an entity might be exposed to, such as their likelihood and their expected Impact on the System (IS), enabling the estimation of the resources necessary to bring the system back into operation. IS corresponds to the reduction of the system’s ability to perform an assigned function after the occurrence of disruptive events. Given this information, the system’s performance should return to its targeted level over time, incurring a Post-interruption Recovery Cost (PCR). In this paper, both IS and PCR are measured as expected costs, weighted with the likelihoods of the disruptive events considered.

The concept of resilience is concerned with the resistance, flexibility and recovery of an entity [5], emphasizing that actions can be undertaken to mitigate IS. A resilient system is defined by the following capabilities: (i) absorption—the capacity to anticipate, minimize and withstand the consequences of disturbances; (ii) adaptation—the capacity for reconfiguration in undesirable situations; and (iii) restoration—the speed and ease with which the system returns to normal operation [5,19–21]. These three capacities make up the “resilience triangle” [5] and should ideally be considered during the design phase of a system to effectively mitigate IS.

**Fig 1:**
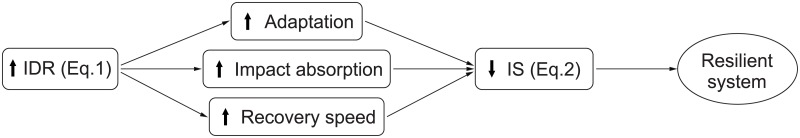
Relationship among IDR, IS, and the three resilience capacities.

This paper focuses on setting a resilience-based strategy that determines the appropriate pre-event actions that have the potential to minimize IS by considering the capacities for resilience previously presented. The investments associated with these three capacities can be defined as Investments in Design for Resilience (IDR), comprising actions undertaken during the system’s design phase that seek to reduce both the impact and the system recovery time, as represented by Fig 1.

As seen in Fig 1, the system designed to absorb and anticipate the impact of an unwanted event and to adapt to new conditions might have a low IS, and thus should be more resilient. In addition, the recovery speed is influenced by the investments to return the system to operation quickly. Thus, this paper aims to demonstrate the important interactions between IDR and IS decisions, in which IDR could positively influence system resilience by increasing absorption and adaption capacities, shortening recovery time and consequently reducing IS.

### Evaluation of critical infrastructures: Resilience vs other concepts

Under uncertainty, a CI can be assessed by different approaches, e.g., resilience [5,19,22], reliability [23,24], risk [25–28], robustness [29,30] and vulnerability [31,32]. According to Hokstad et al. [33], reliability is measured in terms of the probability that a system or a component can perform its required function at a given point of time under a given set of conditions. Traditional risk assessment in turn focuses on the likelihood and consequences of disruptive events, by understanding the nature of potential disturbances, characterizing their negative consequences and mitigating the level of risk which the system is exposed to (e.g., [25,27]). Robustness or vulnerability are often used to measure the extent to which a power grid has high or low reliability [29].

According to Linkov [10], “resilience is not a substitute for principled system design or risk management. Instead, resilience is a complementary attribute that uses strategies of adaptation and mitigation to improve traditional risk management”. Panteli and Mancarella [34] in turn argued that the resilience concept encompasses all of the aforementioned concepts. Indeed, because risk assessment results in an understanding and mitigation of the potential disturbances, and robustness/vulnerability evaluation can help to identify weaknesses and candidates for the implementation of actions of resilience enhancement, these two approaches can serve as inputs to resilience analysis during the CI’s design phase. In contrast, reliability assessment can measure the effectiveness of a resilience-based strategy over time.

Concepts of resilience have been studied regarding infrastructure networks in the areas of supply chain [18,35], transportation systems [36–42], natural gas networks [43], telecommunications [44], water supply networks [45,46] and designs for infrastructure [1,47]. For a comprehensive review of the existing literature on definitions and measures of system resilience, the interested reader can consult Hosseini et al. [48].

In the field of EPSN resilience, there have been papers in the literature with both qualitative [34,49–51] and quantitative [5,52–56] approaches. For example, Panteli et al. [34] evaluated the impact of weather changes on the reliability, operation and resilience of an electric power network by observing the intensity, frequency and duration of severe weather events and proposing plans to increase EPSN resilience. Ouyang et al. [53] used a probabilistic modelling approach to quantify electrical system resilience and economic losses, given the occurrence of hurricanes, assessing (i) hurricane risk, (ii) fragility, (iii) performance and (iv) restoration. Kim et al. [54] investigated the topological properties of the South Korean Power Grid (KPG), including its resilience. Their study considered node-based and network-based measures to characterize the structural dimensions of a network and to understand its topology and resilience. The results obtained concerning the KPG were compared with random and scale-free reference networks. Finally, several suggestions were made to improve its resilience. Fang et al. [55] considered investments in capacity expansion and backup to evaluate the performance of electrical transmission networks under nominal operations and after deliberate attacks. Dewenter et al. [56] studied the resilience of power-flow models to the failure of a transmission line, with resilience characterized in terms of the “backup capacity”, defined as the additional capacity of the links that must be supplied to secure stable operation of the link with the greatest load in case of an attack or a failure in that link.

According to Cuadra et al. [29], there are two different approaches to evaluating power grid resilience. The first is solely based on topological concepts, using metrics such as the mean path length, clustering coefficients, efficiency and betweenness centrality [57,58]. The second, a hybrid approach, introduces some electrical engineering concepts in an effort to enhance the topological approach, using metrics such as electrical betweenness and net-ability [56,59–62]. For example, Guohua et al. [59] presented an assessment of the North China power grid based on complex network theory to investigate the tolerance of the power grid to attacks. Pepyne et al. [62] evaluated the resilience of a synthetic Watts-Strogatz network with 200 nodes and 400 links in terms of link attack schemes, disruption of the network and overhead lines.

Due to the increased focus on structural dimensions of resilience, a limited number of quantitative studies focusing on resilience and the variables that affect system performance, such as the cost of post-disruption operation, customer service and investments in design (Dixit et al. [63]). Therefore, the present work aims to fill this gap by assessing the resilience of power grids in meeting customer demand, not only by designing a system with increased resilience, but also by identifying how much resilience is improved when considering different possible methods to invest in the design of a system.

Furthermore, even with the variety of applications of resilience, to the best of the authors’ knowledge, the aforementioned articles do not consider the impact of disruptions to the electricity supply on industrial clients. Indeed, most of the resilience literature has overlooked differences among customers and their needs. Thus, our goal is to assess power grids’ resilience with a focus on the industrial client perspective (Kwasinski [64]). Therefore, the proposed framework is intended to establish a “view of the grid” from the perspective of an industrial client; thus, our focus is not to address different types of failures in the main electrical power grid but to improve the resilience of the power supplies connected to industrial clients.

In this context, the main contribution of our work is to propose an optimization model using MILP to make decisions related to investments in the design of power grid resilience with a focus on the customer perspective. Our paper evaluates how costs associated with investments in the design phase can reduce both the impact and recovery efforts over time, given the occurrence of an undesired event. In other words, we can now determine how financial resources should be spent to design a resilient power grid. In this manner, we provide a glimpse into the decisions that consumers of electric power can make that influence the resilience of the overall system.

Additionally, in contrast to [29,41,53–62,64], this article evaluates the performance of the electricity supply over time by examining the evolution of the impact of disruptive events on the system and its response. Despite the importance of considering this factor, the vast majority of work on power grids has not included the time dimension in its analyses of resilience [5,65,66].

## The proposed approach

### Scope of the analysis

In this section, we first describe the main characteristics of an electrical grid to contextualize the scope of our analysis. The bulk power system is generally designed in accordance with the N-1 security criterion, requiring the system be able to bear the loss of one major component (mainly transmission lines and power transformers) without interrupting the electricity supply [33]. Moreover, typical distribution networks usually have interconnected feeders that can be automatically and/or manually switched on in case of failures.

**Fig 2:**
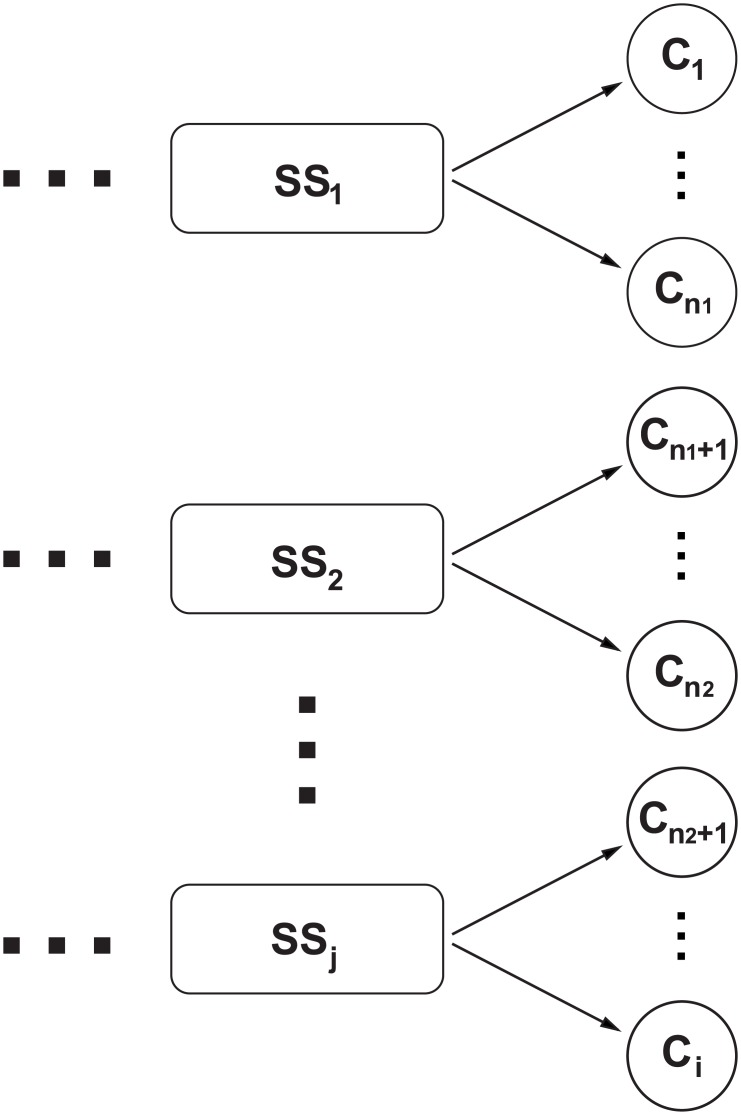
Representation of the electrical connections for industrial clients (C1, …, Ci). Industrial customers, along with nodes and links, comprise the power grid portion considered in the proposed model and analyses.

In contrast, the electric power supply to industrial clients is usually provided by a single connection line and a step-down substation, and failures in this infrastructure can cause power supply interruptions and therefore additional production costs. Given this fact, the scope of our analysis is highlighted in Fig 2, representing our focus on the user perspective. Thus, disruptions of the system are analysed in terms of interruptions of the electricity supply to industrial clients that, for instance, serve critical societal functions.

Fig 2 contains a set of Subtransmission Substations (SSs) denoted by SS_j_, which are responsible for ensuring energy supply to industrial clients, denoted by C_i_, where i > n_2_ + 1 >…n_1_ > 1, through subtransmission lines. Under normal conditions, each client has a demand Q_i_, which is served by a specific SS_j_ (primary assignment) with capacity K_j_ to accommodate the demand assigned to it. The electrical connection of industrial clients shown in Fig 2 could be generalized to other configurations. For example, it could involve a different number of SSs or industrial clients, which would only require modifying the allocation of clients per SS.

In this manner, the proposed model provides some alternatives to improve the resilience of the electric power supply for industrial plants, including normally open backup power lines, active parallel lines, purchasing of diesel generators, or increases in restorative capacity. However, the implementation of these reinforcements, in practice, depends on the costs of expanding the electrical connection and the expenditures arising from interruptions to the energy supply. Thus, based on some input data regarding a set of industrial plants, the solution proposed by the model indicates whether these alternatives should be implemented.

### Modelling assumptions

This paper proposes an optimization model to minimize the total expected costs by means of implementing resilience-based alternatives that are useful in case of stoppages of the supply of electrical energy to industrial clients due to disruptions in the configuration analysed in Fig 2. The stochastic characteristic of the proposed model relies on considering different disruptive scenarios (each with its own probability of occurrence) in the electrical connections to industrial clients; the probabilities of occurrence are used in the calculation of the total expected cost.

However, there are myriad events that might cause disruptions in the electricity supply, for example, climate change [67], natural disasters [51,68], physical attacks [69] and terrorism [50]. However, this paper does not intend to consider every possible contingency or to model the causes of the disruptive events that affect power supplies to industrial clients. To evaluate different scenarios of disruption, we consider the following assumptions for the power grid in Fig 2:

- SS_j_ can be affected by an event that will partially or fully impact its capacity, thereby influencing the supply of the set of customers C_i_ assigned to it. This capacity will be recovered over time in accordance with the recovery rate of the system;
- The subtransmission line between SS_j_ and a connected C_i_ can be affected, thus halting only the supply of C_i_;
- Multiple failures can occur, affecting SS_1_ and SS_2_, two subtransmission lines, SS_j_ and a subtransmission line not connected to it, or a subtransmission line and its corresponding SS_j_.

Thus, the method will determine the optimal allocation of resources to minimize the overall expected costs for designing this power grid, assuming that an undesirable scenario could occur. In addition to the post-event response (i.e., efforts to restore the supply of energy to industrial plants), we consider pre-event decisions related to investments in improving resilience, which can be accomplished by including absorptive, adaptive and restorative capacities [1,5,11] in the phase of designing the electrical connections to industrial customers. The idea is to incorporate the concept of resilience into the design of the system, thereby considering different possibilities of IDR and the respective IS and PCR.

This problem gives rise to an MILP approach, for which the parameters and variables are described in Tables 1 and 2, respectively. The binary variables are set so that 1 indicates the existence or operation of some SS or link of the system and 0 otherwise.

**Table 1:** Description of the parameters.

Parameter	Description	Range or unit
T	Time period	Hours
d	Deadline to restore system to nominal performance threshold	Hours
*ℓ*	Recovery rate of subtransmission lines	Line/hour
Q_i_	Demand of C_i_ per period	MVA/hour
r	Recovery rate of SS capacity	MVA/hour
G	Capacity of diesel generator	MVA
K_j_	Initial capacity of SS_j_	MVA
p_c_	Probability of occurrence of scenario c	[0,1]
V_jc_	Portion of SS_j_ capacity affected in scenario c	[0,1]
F_ijc_	Occurrence of an event in the subtransmission line between SS_j_ and C_i_ in scenario c	0 or 1
L_ij_	Predefined connections between SS_j_ and C_i_	0 or 1
α_j_	Cost of adding K_j_ units to SS_j_ capacity	$
λ_ij_	Cost of establishing a backup (SS_j_ for C_i_)	$
φ_ij_	Cost of adding a redundant line between SS_j_ and C_i_	$
γ	Cost of siting a generator	$
μ	Cost of adding resources to accelerate SS recovery	$
ρ	Cost of supplying demand	$
θ	Cost of supplying demand by means of a generator	$
ϕ_i_	Penalty for unmet demand of C_i_	$
δ_i_	Penalty for unmet demand of C_i_ after a deadline d	$
ω_i_	Penalty for unmet demand of C_i_ when C_i_ has diesel generators	$
π	Cost of recovering SS capacity	$
σ	Cost of recovering subtransmission lines	$
M	Budget available for costs incurred for IDR and PCR	$

**Table 2:** Description of the variables.

Variable	Description	Range or unit
A_i_	Addition of K_j_ units to the capacity of SS_j_	MVA
W	Additional resources for SS recovery	MVA/period
n_i_	Quantity of generators added to C_i_	units
B_ij_	Backup connection between C_i_ and SS_j_	0 or 1
H_ij_	Redundant line between C_i_ and SS_j_	0 or 1
g_itc_	Operation of C_i_ generators in period t for scenario c	0 or 1
S_ijtc_	Operation of the subtransmission system from SS_j_ to C_i_ in period t for scenario c	0 or 1
O_ijtc_	Operation of the subtransmission line between SS_j_ and C_i_ in period t for scenario c	0 or 1
U_itc_	Capacity of SS_j_ in period t for scenario c	MVA
R_jtc_	Capacity of SS_j_ recovered in period t for scenario c	MVA
x_ijtc_	Portion of C_i_ demand supplied by SS_j_ in period t for scenario c	[0,1]
z_itc_	Portion of C_i_ demand supplied by generators in period t for scenario c	[0,1]
D_itc_	Portion of C_i_ demand supplied in period t for scenario c	[0,1]
y_itc_	Portion of C_i_ demand that is not met in period t for scenario c when C_i_ does not have a generator	[0,1]
h_itc_	Portion of C_i_ demand that is not met in period t for scenario c when this client uses its diesel generator	[0,1]
N_itc_	Portion of C_i_ demand that is not met in period t for scenario c	[0,1]
a_ijtc_	Portion of the subtransmission line between SS_j_ and C_i_ recovered in period t of scenario c	[0,1]

### Design phase: Pre-event costs

The options available for pre-event investments are translated into costs defined as IDR, and they are divided into three types of capacity: adaptation, absorption and restoration. Considering possible system interruptions and according to the adaptive concepts presented in [1,5], the possibilities for increasing the adaptive capacity are the following.

- To establish a backup line between SS_k_ and C_i_ so that the impact on the industrial plant operation will be reduced. Indeed, if SS_j_ is affected, its demand can be supplied by SS_k_, with k ≠ j. Determining which SS_k_ would work as a backup for C_i_ will be based on the cost to establish the new connection. Backup lines are deemed to operate in hot standby mode.
- To build a redundant line that shares a load with the main line (active parallel) to ensure the supply of C_i_ from its corresponding SS_j_. The model will determine the existence (or not) of this line so that, if the main line is affected, the redundant one will be able to support the full load.
- To invest in diesel generators to keep the plant at partial or full operation until the main power supply returns. Failures on demand of the diesel generators are not considered here.

Investments in absorptive capacity can be made by expanding the capacity of SS_j_ so that the system will be able to better respond to an event that could affect subtransmission sub-stations or links. The opportunity to invest and expand the capacity of each SS_k_ allows the system to more easily bear the loss of one or more SS_j_ (k ≠ j) because the system will have additional capacity to manage the additional demand of SS_j_, and consequently will continue to meet demands (partially or totally). The investments in restorative capacity will be spent on deploying additional maintenance crews and buying spares to increase the recovery rate. Considering the available options, the IDR can be expressed as shown in Eq (1):

$$IDR\;=\;\sum_{j}\alpha_{j}A_{j}+\;\gamma \sum_{i}n_{i}+\;\sum_{i}\sum_{j}\lambda_{ij}B_{ij}+\;\varphi \sum_{i}\sum_{j}H_{ij}+\;\mu w$$ (1)

The first part of Eq (1) corresponds to investing in absorption, which is the possibility of adding capacity to each SS_j_. The next three terms correspond to possible investments in adaptive capacity: installing generators for C_i_, establishing backups for clients so their demands can be met by another SS (besides their primary supplier) and the possibility of setting a redundant line between C_i_ and SS_j_, respectively. The last term corresponds to the investment in increasing the recovery rate.

### Post-event costs

Post-event expected costs are associated with the financial impact of IS caused by a disruptive scenario on system performance and the efforts (PCR) to restore the system supply capacity. We consider that the losses of industries are a step-change function of the demand that is not met in period t for scenario c and for each type of client. However, there is a monetary penalty for each unmet MVA.

We also consider that industrial plants manufacture products, which have different added values; thus, the penalty depends on the specific industrial sector. Therefore, IS can be specified as the impact on the demand supply, and it is expressed in Eq (2):

$$IS\;=\;\sum_{c}p_{c}[\rho \sum_{i}Q_{i}\sum_{j}\sum_{t}x_{ijtc}+\;\theta \sum_{i}Q_{i}\sum_{t}z_{itc}+\sum_{i}\phi_{i}Q_{i}\sum_{t}y_{itc}+\sum_{i}\delta_{i}Q_{i}\sum_{\tau \;=\;d}^{T}y_{i\tau c}+\sum_{i}\omega_{i}Q_{i}\sum_{t}h_{itc}+\sum_{i}\delta_{i}Q_{i}\sum_{\tau \;=\;d}^{T}h_{i\tau c}]$$ (2)

where p_c_ is the probability of each scenario c, which corresponds to a disturbing event that causes an interruption to the energy supply. The first and second terms of Eq (2) represent the cost of supplying power from SS_j_ and diesel generators, respectively. The third part reflects the penalty incurred because the main SS did not meet some portion of clients’ demands. The fourth portion represents an additional fee for unmet demand beyond deadline d, which is usually established in the contract signed with the client. In this manner, if the supplier fails to meet such a time limit, there will be additional costs in addition to the existing penalties. Despite its importance, this penalty structure is not considered in the works mentioned above.

The fifth term corresponds to the penalty for not meeting some portion of clients’ demands when these clients have diesel generators. However, as before, there is a possible sixth term, which is an additional fee that is charged if the non-supply of power extends beyond d. We considered the fifth and sixth parts of Eq (2) because of the specific characteristics of the production processes. Usually, industries suffer great losses due to failures in the power supply even if interruptions are short. Equipment such as reactors, homogenizers, blast furnaces and other critical items do not simply return to their operational state when the power supply is re-established, related to the inputs not being processed by the equipment (work-in-process) due to interruptions in the supply of power, which cannot usually be made to the full specifications set. This failure indicates that there has been a lack of control in the process. Moreover, even when the energy returns, there are production losses until the process returns to the default condition.

Therefore, the possibility of using a diesel generator can reduce the impacts caused by this problem and keep the equipment in operation to remove, for example, the material in process until power is restored, thus reducing the costs incurred by this interruption. In this case, the plant would be penalized only with the loss of production during this period and would no longer suffer losses due to the time spent on re-establishing process control. Therefore, the penalties that might be associated with the lead time when the power supply is cut and the generators are started will not be considered. Note that this approximation is reasonable given that the generators are equipped with automatic start, which usually takes 10 to 30 seconds to become operational.

PCR, in turn, includes the costs associated with the resources required to recover the system due to disturbances, i.e., the cost of restoring the performance of the system after an interruption *c*. The expected PCR is shown in Eq (3):

$$PCR\;=\;\sum_{c}p_{c}[\pi \sum_{j}\sum_{t}R_{jtc}+\sigma \sum_{i}\sum_{j}\sum_{t}a_{ijtc}]$$ (3)

where the first part indicates the costs associated with the use of recovery resources, if the recovery actions are directed to SS_j_, and the second term represents the cost associated with recovering a subtransmission line between SS_j_ and C_i_.

### Formulation of the model

The stochastic optimization model proposed is defined as an MILP problem with an objective function that combines the cost of investing in resilience-based actions in the network design phase (IDR) and the expected costs related to system performance and recovery (IS plus PCR). Thus, the objective function (Eq (4)) is the sum of IDR, IS and PCR, which are presented in Eqs (1), (2) and (3), respectively.

$$\begin{array}{l} \text{Min}\;\text{IDR}+\text{IS}+\text{PCR}=\;\sum_{\text{j}}\alpha_{\text{j}}{\text{A}}_{\text{j}}+\;\gamma \sum_{\text{i}}{\text{n}}_{\text{i}}+\;\sum_{\text{i}}\sum_{\text{j}}\lambda_{\text{ij}}{\text{B}}_{\text{ij}}+\;\varphi \sum_{\text{i}}\sum_{\text{j}}{\text{H}}_{\text{ij}}+\;\mu \text{w}+\;\sum_{\text{c}}{\text{p}}_{\text{c}}[\rho \sum_{\text{i}}{\text{Q}}_{\text{i}}\sum_{\text{j}}\sum_{\text{t}}{\text{x}}_{\text{ijtc}}+\\ \theta \sum_{\text{i}}{\text{Q}}_{\text{i}}\sum_{\text{t}}{\text{z}}_{\text{itc}}+\sum_{\text{i}}\phi_{\text{i}}{\text{Q}}_{\text{i}}\sum_{\text{t}}{\text{y}}_{\text{itc}}+\sum_{\text{i}}\delta_{\text{i}}{\text{Q}}_{\text{i}}\sum_{\tau =\text{d}}^{\text{T}}{\text{y}}_{\text{i}\tau \text{c}}+\sum_{\text{i}}\omega_{\text{i}}{\text{Q}}_{\text{i}}\sum_{\text{t}}{\text{h}}_{\text{itc}}+\sum_{\text{i}}\delta_{\text{i}}{\text{Q}}_{\text{i}}\sum_{\tau =\text{d}}^{\text{T}}{\text{h}}_{\text{i}\tau \text{c}}+\;\pi \sum_{\text{j}}\sum_{\text{t}}{\text{R}}_{\text{jtc}}+\\ \sigma \sum_{\text{i}}\sum_{\text{j}}\sum_{\text{t}}{\text{a}}_{\text{ijtc}}] \end{array}$$ (4)

subject to:

$$\sum {}_{\text{j}}{\text{B}}_{\text{ij}}\;\leq \;1\;\forall \text{i}$$ (5)

$$D_{itc}\;=\;\sum_{j}x_{ijtc}+\;z_{itc}\;\forall i,t,c$$ (6)

$$S_{ijtc}-x_{ijtc}\;\geq 0\;\forall i,j,t,c$$ (7)

$$g_{itc}-\;z_{itc}\geq 0\;\forall i,t,c$$ (8)

$$z_{itc}\;Q_{i}\leq \;n_{i}\;G\;\forall i,t,c$$ (9)

$$\sum_{i}x_{ijtc}Q_{i}\;\leq \;U_{jtc}\;\forall j,t,c$$ (10)

$$N_{itc}\;=\;y_{itc}+\;h_{itc}\;\forall i,t,c$$ (11)

$$g_{itc}-\;h_{itc}\;\geq 0\;\forall i,t,c$$ (12)

$$g_{itc}+\;y_{itc}\;\leq 1\;\forall i,t,c$$ (13)

$$D_{itc}+\;N_{itc}\;=\;1\;\forall i,t,c$$ (14)

$$g_{itc}\;\leq \;n_{i}\;\forall i,t,c$$ (15)

$$S_{ijtc}\leq \;L_{ij}H_{ij}+\;O_{ijtc}+B_{ij}\;\forall i,j,t,c$$ (16)

$$U_{jtc}-K_{j}\;S_{ijtc}\;\geq 0\;\forall i,j,t,c$$ (17)

$$O_{ijtc}\leq (1-F_{ijc})L_{ij}+\sum_{\tau \;=\;1}^{t-1}a_{ij\tau c}\;\forall i,j,t,c$$ (18)

$$O_{ijTc}\leq L_{ij}\;\forall \;i,j,c$$ (19)

$$O_{ijTc}\;=\;L_{ij}\;\forall \;i,j,c$$ (20)

$$U_{jtc}\;=\;(1-\;V_{jc})(K_{j}+{K_{j}A}_{j})+\;\sum_{\tau \;=\;1}^{t-1}R_{j\tau c}\;\forall j,\;t,c$$ (21)

$$U_{jtc}\;\leq \;K_{j}+\;K_{j}A_{j}\;\forall j,t,c$$ (22)

$$U_{jTc}\;=\;K_{j}+\;K_{j}A_{j}\;\forall j,c$$ (23)

$$\sum_{j}R_{jtc}\;\leq \;r+\;w\;\forall t,c$$ (24)

$$\sum_{i}\sum_{j}a_{ijtc}\leq \ell \;\forall t,c$$ (25)

$$\alpha \sum_{j}A_{j}+\;\gamma \sum_{i}n_{i}+\;\sum_{i}\sum_{j}\lambda_{ij}B_{ij}+\;\varphi \sum_{i}\sum_{j}H_{ij}+\;\mu w+\;\sum_{c}p_{c}[\pi \sum_{j}\sum_{t}R_{jtc}+\sigma \sum_{i}\sum_{j}\sum_{t}a_{ijtc}]\leq M$$ (26)

$$A_{j},\;n_{i},\;w,\;R_{jtc},\;U_{jtc}\;\geq 0$$ (27)

$$x_{ijtc},\;z_{itc},\;y_{itc},\;h_{itc},a_{ijtc},\;D_{itc},\;M_{itc},\;O_{ijtc}\geq 0$$ (28)

$$B_{ij},\;H_{ij},\;S_{ijtc},\;g_{itc}\;\in \;\{0,1\}$$ (29)

Constraint 5 is related to the limit of connections per client, assuming that each client can have only one backup connection at most. We assume this limit because (i) the cost of implementation of a backup power line is higher than that of a diesel generator; (ii) multiple backup lines would require increased space, which is not always feasible, mainly near urban areas; and (iii) finally, provided that the substation is operational, a single line would provide all of the energy needed to supply the industrial plant, while it would require multiple generators to have the same outcome.

Constraints 6–14 are associated with meeting the clients’ demand. The demand of each client can be served by the corresponding SS_j_ and its diesel generators (Constraint 6) so that the demand of C_i_ can only be served by SS_j_, assuming this link exists and is operational (Constraint 7). Therefore, the portion of C_i_ demand served by generators can only exist if generators have been installed in C_i_ (Constraint 8), and this amount cannot exceed the capacity of the generators (Constraint 9). In addition, the whole demand that SS_j_ is expected to meet cannot exceed its capacity (Constraint 10). Constraint 11 represents the portion of C_i_ demand that is not supplied in each period, which can occur if either SS or the generators do not have sufficient capacity. If C_i_ generator is activated, information represented by g_itc_, the unmet portion of C_i_ demand is represented by h_itc_ (Constraint 12); otherwise, it will be represented by y_itc_ (Constraint 13). Consequently, the portion of each client’s demand that is met and the portion that is not met in each period are complementary factors (Constraint 14).

Generators can only be activated if the subtransmission system for C_i_ has been affected, given that the investment in their acquisition has been made (Constraint 15). In this context, the predefined subtransmission system operates in series such that, if any component that provides energy for C_i_ is affected, the power does not reach C_i_. Therefore, Constraints 16 and 17 correspond to the connection between SS_j_ and C_i_ in accordance with the operational condition of each component of this system. The connection is operational if and only if at least K_j_ of the capacity of SS_j_ has been recovered (Constraint 17). Moreover, Constraint 16 represents the operation of the connection between SS_j_ and C_i_, considering that the following:

1. If C_i_ is primarily connected to SS_j_, this connection might or might not be operational (O_ijtc_);
2. If C_i_ is primarily connected to SS_j_, this connection could be ensured by a redundant line (H_ij_); and
3. If C_i_ is not primarily connected to SS_j_, SS_j_ might be its backup (B_ij_).

Constraints 18–20 register the state (whether operational or not) of the subtransmission line between SS_j_ and C_i_, given that it is a primary connection (Constraint 19), and this line is subject to the occurrence of events that can affect its performance. A portion of each line (a_ijtc_) can be recovered in each period and for a given scenario using the recovery rate *ℓ* and these lines must be fully recovered over time (Constraints 18 and 20) using the available resources (Constraint 25), which are shared among all subtransmission lines.

Constraints 21–23 represent the determination of the capacity of SS_j_, given that an event affects its operation, and its capacity must be recovered over time. Immediately after the occurrence of the disruptive event, SS_j_ has reduced capacity or no capacity at all. Thus, restoration efforts can be undertaken by increasing capacity by r (the recovery rate parameter in MVA/hour). This process continues, with recovery efforts being made hourly so that the entire capacity is recovered until T is reached. In the model, the SS recovery rate can be increased using additional resources (variable w), which should be devoted to hiring maintenance crews and buying spares.

Constraint 24 corresponds to the total resources available to recover SS and must be shared among all SSs. In addition, the costs associated with IDR and PCR cannot exceed the limit M, as shown in Constraint 26, which represents financial constraints. Constraints 27–29 specify the variation ranges of the variables as being non-negative integer, non-negative real and binary, respectively.

We demonstrate the applicability of the proposed model. Our aim is to evaluate how the strategies for improving resilience vary for a wide range of scenarios and for different investment options, assessing the corresponding impacts over time. In addition, the example is useful for discussing the validation and verification of the model.

### Application example

#### Description of the problem

**Fig 3:**
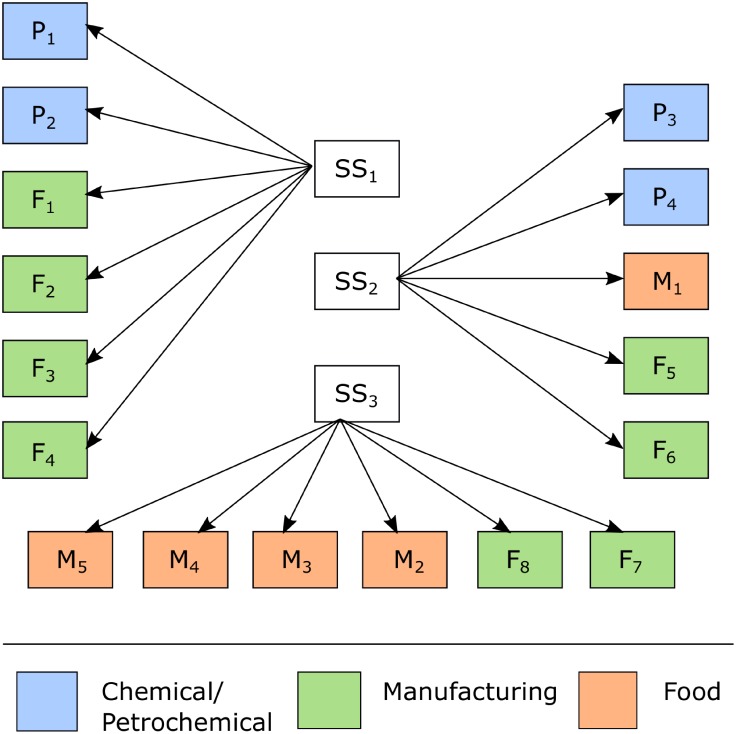
Representation of power grid supply to industrial clients.

This section discusses the application of the proposed model to an example involving an EPSN with industrial clients from the chemical/petrochemical, food and manufacturing sectors. As mentioned above, this paper does not aim to consider every possible contingency over the whole power supply network. In fact, our aim is to improve the resilience of the power supply with regard to industrial clients’ connections to the electrical power grid. This situation is of practical application for medium to large industries that have very high costs (and thus very low tolerance) when interruptions to the power supply occur in their production plants. Therefore, alternatives that improve the resilience of industrial clients’ connections to the EPSN are provided. Fig 3 shows the power grid that will be addressed in this section. In Fig 3, clients are represented according to their sectors.

In this example, the power grid consists of 3 substations that together supply 150 MVA (Table 3) to industrial customers such that the capacity of each SS is given by the total demand assigned to it. Having both the added value of the products and the eventual loss of production as criteria, the chemical/petrochemical, manufacturing and food industries are ranked in this order, according to their level of importance to local economic activity. Thus, the energy supplier incurs different penalties for demand not supplied because of a disruption in the performance of the system.

**Table 3:** Client data.

Client	Industrial segment	Demand (MVA)
P_k_, with k = 1, 2, 3, 4.	Petrochemical/chemical	15
M_m_, with m = 1, 2, 3, 4, 5.	Manufacturing	10
F_r_, with r = 1, 2, 3, 4, 5, 6, 7, 8.	Food	5

To show how disruptions in the network can affect the investments necessary to achieve an optimal, resilient design, we defined a set of scenarios and their associated probability p_c_. These scenarios are used to specify the loss of SS supply capacity and the loss of subtransmission lines between SS and its clients.

As discussed above, interruptions can occur due to internal or external factors, including various natural factors. For example, in Brazil, atmospheric discharges and torrential rains, combined with falling trees, can interrupt the power supply to industrial clients. According to [30], the disaster probabilities are difficult to quantify. However, for this example, we are not concerned with identifying and analysing specific causes of events that could affect the network. In fact, our aim is to quantify several ways by which the system might become unavailable.

In this context, the proposed method for defining p_c_ considers the observation of the network as a random experiment, for which three possible situations can arise: (i) no occurrence of a disruptive event; (ii) a single failure; or (iii) multiple failures. A single failure is understood as the loss of a node (SS) or a link (subtransmission lines). Multiple failures can be observed in (i) simultaneous failures: SS_1_ and SS_2_, two subtransmission lines and SS and a subtransmission line not connected to it; or (ii) cascading failures since failures in both the line and its respective SS are a sort of cascading failure and cannot be considered independent events. Thus, the costs related to their recovery should also be considered. Observations of three or more simultaneous failures are not considered because they are very unlikely to occur.

We also consider simultaneous failures in both SS_1_ and SS_2_ because they are assumed to be connected to the same step-down Transmission Substation (TS). Thus, this failure could be related to a common cause, such as the loss of TS supply. However, we do not consider other joint failures of SS because they are very unlikely to occur, especially if they are connected to independent TSs. Table 4 shows that each element of the sample space (Ω) is related to a scenario, which represents how an undesired event can impact the supply of electricity to industrial clients; all scenarios are assumed to be mutually exclusive.

**Table 4:** Description of the scenarios.

Type of event	Description	p_c_	Number of similar scenarios
Single failures	{S_j_}: failure of the j-th SS with j = 1, 2, 3;	c_1_x	3
	{LP_k_} failure of the line between the k-th chemical/petrochemical and the corresponding SS;	c_2_x	4
	{LM_m_}: failure of the line between the m-th manufacturer and its SS	c_2_x	5
	{LF_r_}: failure of the line between the r-th food and its SS.	c_2_x	8
Multiple failures	{S_1_S_2_}	X	1
	{S_j_LP_k_}, k not connected to j	c_1_c_2_x^2^	8
	{S_j_LM_m_}, m not connected to j	c_1_c_2_x^2^	10
	{S_j_LF_r_}, r not connected to j	c_1_c_2_x^2^	16
	{S_j_LP_k_}, k connected to j	c_1_x	4
	{S_j_LM_m_}, m connected to j	c_1_x	5
	{S_j_LF_r_}, r connected to j	c_1_x	8
	{LP_k_LP_q≠k_}	$c_{2}^{2}x^{2}$	6
	{LM_m_LM_q≠m_}	$c_{2}^{2}x^{2}$	10
	{LF_r_LF_q≠r_}	$c_{2}^{2}x^{2}$	28
	{LP_k_LM_m_}	$c_{2}^{2}x^{2}$	20
	{LP_k_LF_r_}	$c_{2}^{2}x^{2}$	32
	{LM_m_LF_r_}	$c_{2}^{2}x^{2}$	40
{No failure}	No occurrence of a disruptive event	$1\;-x(1+20c_{1}+17c_{2})-x^{2}(34c_{1}c_{2}+136c_{2}^{2})$	1

Furthermore, scenario {S_1_S_2_} (related to a TS failure) is considered less likely than joint failures {S_j_LP_k_}, {S_j_LM_m_} and {S_j_LF_r_} with k, m and r connected to j, which in turn are considered as probable as {S_j_}. Additionally, {S_j_} is less likely than scenarios {LP_k_}, {LM_m_} and {LF_r_}, which represent the disconnection of single lines. Such an assumption is based on the practice that a TS is designed with a more robust bus or better switching schemes, compared to an SS [70].

Given this assumption, we can establish relationships among the probabilities of occurrences of these scenarios. More specifically, if x is the probability of the scenario {S_1_S_2_}, then P({S_j_LP_k_}) = P({S_j_LM_m_}) = P({S_j_LF_r_}) = P({S_j_}) = c_1_x for k, m and r connected to j; and P({LP_j_}) = P({LM_m_}) = P({LF_r_}) = c_2_x, where c_1_ and c_2_ are positive constants such that c_1_ < c_2_.

Moreover, the probabilities of the scenarios with simultaneous failures (except {S_1_S_2_}, {S_j_LP_k_}, {S_j_LM_m_} and {S_j_LF_r_} with k, m and r connected to j and with the corresponding probabilities defined above) are given by multiplying the probabilities of their respective single scenarios. For example, if c_2_x is the probability of {LP_k_}, then the probability of {LP_k_LP_q≠k_} is $c_{2}^{2}x^{2}$. In this manner, Table 4 shows the scenarios and their respective probabilities. Note that scenarios {S_1_}, {S_2_} and {S_3_} are equally likely. Thus, scenario type {S_j_}, j = 1, 2, 3, represents three different scenarios with similar definitions and likelihoods (each corresponding to the failure of one SS). The number of similar scenarios is also indicated in Table 4, which shows a total of 209 possible scenarios. Then, the event “no occurrence of a disruptive event (no failure)” is considered complementary to the other failure scenarios.

The consequences of each of these scenarios are different; for example, scenarios {LP_1_LP_2_} and {LP_2_LP_3_} are equally likely, but their effects can differ because P_1_ and P_2_ are connected to SS_1_, whereas P_3_ is connected to SS_2_. Thus, all scenarios should be incorporated into the optimization problem.

In this context, we analysed four cases, for each of which all of the scenarios shown in Table 4 were considered. The different cases were defined based on the probability of the scenario {no failure}. Thus, x is estimated by the definition of the probability of {no failure} and using the property that the sum of probabilities of all scenarios equals 1. For the positive constants c_1_ and c_2_ with 0 < c_1_ < c_2_, the computation of x is always possible. Therefore, having obtained x, the probability of the other scenarios can be estimated using the relations given in Table 4.

We cannot predict exactly which adverse events will occur or when and with what intensity. Nevertheless, given that our approach anticipates the resilience pre- and post-event actions that should be considered, using the probabilities of disruptive events is a method to represent their intrinsically uncertain nature, and doing so also permits the calculation of the expected cost, which is a measure that can guide how resources should be allocated to enhance resilience. In the next section, we present examples of applying the proposed model, which was solved using IBM ILOG CPLEX software, which applies the exact Branch-and-Cut technique (Hillier and Lieberman) [71].

## Results and discussion

The probability of scenario {no failure} and the corresponding x for each of the 4 cases are shown in Table 5. Note that we consider P{No failure} = 0.9, 0.7, 0.3, 0.0 for cases 1, 2, 3 and 4, respectively. In other words, we assume that the probability of a disruptive event is low in case 1. Next, we increase this probability in cases 2 and 3. Finally, we analyse in case 4 a situation in which a disruption will occur for certain. These cases were defined to evaluate the behaviour of the system over T = 8 hours and the response of the model to different possibilities. However, we considered c_1_ = 10 and c_2_ = 100, i.e., the failure of a subtransmission line is ten times more likely than the failure of an SS or of a line and its respective SS.

**Table 5:** Probability of cases 1, 2, 3 and 4.

	Probabilities of Cases			
	1	2	3	4
{No failure}	0.9	0.7	0.3	0
x	5.10E-05	1.43E-04	3.02E-04	4.05E-04

The results presented in this section were obtained disregarding financial resource constraints. In fact, we disregard Constraint 26 to achieve an optimal resilience strategy with unconstrained financial resources. We also perform sensitivity analysis to assess the impact of limited budgets on the optimal resilience strategy and hence on system performance (see next section). The parameter values for the proposed model shown in Table 6 are fictitious for the sake of confidentiality. However, they were carefully estimated to represent reality.

**Table 6:** Values of the parameters used for all 4 cases.

Parameter	Description	Value
α_j_	Cost of adding Kj MVA of capacity to SS	$ 3 million
λ_ij_	Cost of establishing backup (SSj for Ci)	k$ 480
φ	Cost of adding a redundant subtransmission line (SSj for Ci)	k$ 350
γ	Cost of installing a diesel generator	k$ 260
*μ*	Cost of adding resources to accelerate SS recovery	k$ 100 /MVA
ρ	Cost of meeting the demand for supply (from the main power supply system)	$ 0.5 /MVA
θ	Cost of meeting the demand for supply from the diesel generator	$ 0.8 /MVA
ϕ_*i*_	Penalty for unmet demand of Ci	k$ 200 / MVA (chemical/petrochemical), k$ 180 / MVA (manufacturing), k$ 160 / MVA (food)
δ_i_	Penalty for unmet demand of Ci, after a certain deadline d	k$ 200 / MVA (chemical/petrochemical), k$ 180 / MVA (manufacturing), k$ 160 / MVA (food)
ω_i_	Penalty for unmet demand of Ci, when Ci has generators	k$ 100 /MVA (chemical/petrochemical), k$ 80 /MVA (manufacturing), k$ 60 /MVA (food)
π	Cost of recovering SS capacity	k$ 15 /MVA
*σ*	Cost of recovering subtransmission lines (between SSj and Ci)	k$ 50
T	Time period	8 hours
d	Deadline for return of subtransmission to normal operation	3 hours
r	Recovery rate for SS capacities	20 MVA/hour
G	Capacity of a diesel generator	2 MVA
*ℓ*	Recovery rate for subtransmission lines	0.5 line/hour

**Fig 4:**
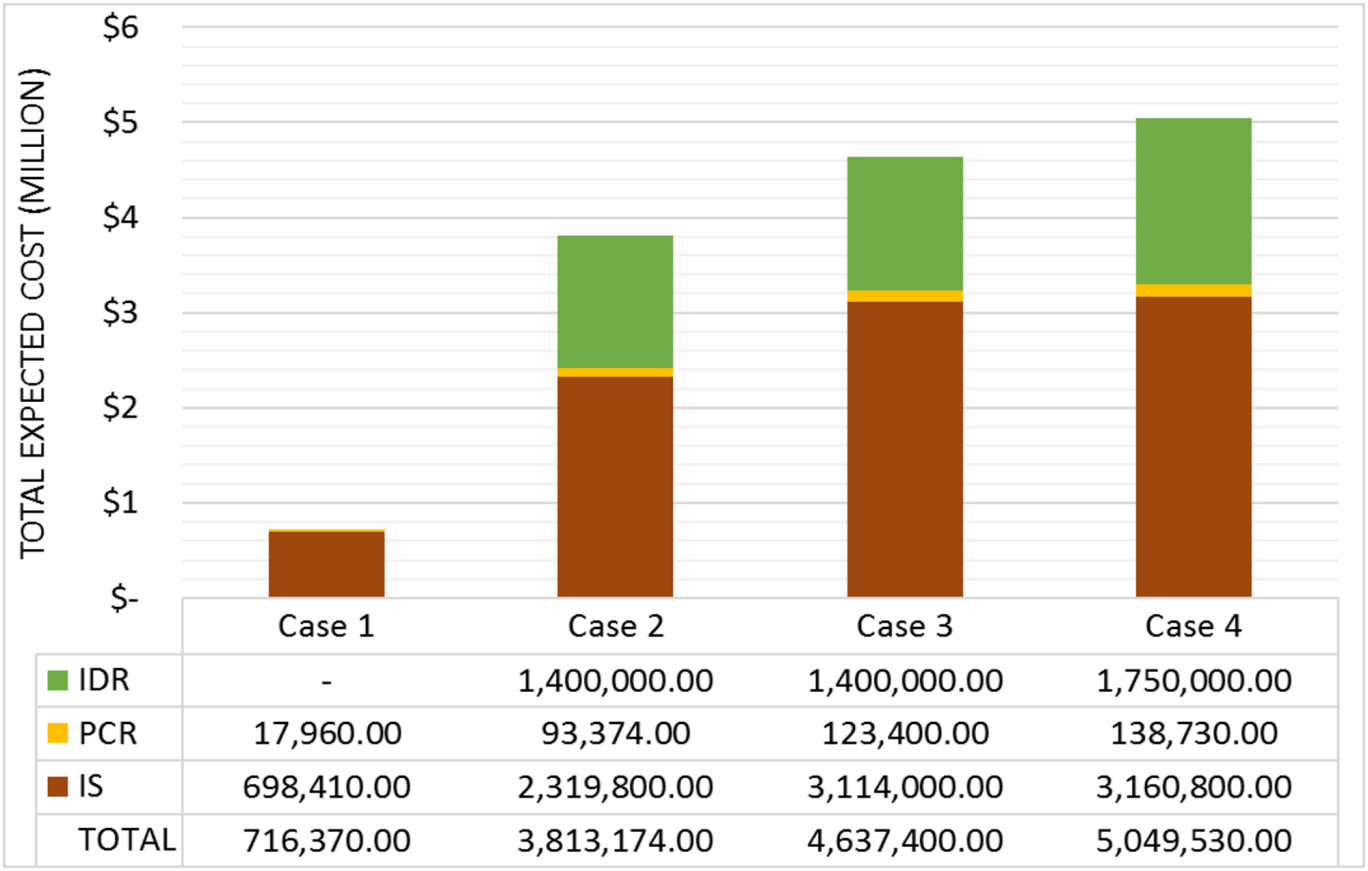
Total expected costs of the 4 different cases.

The comparison between the results in terms of IS, IDR and PCR obtained for each of the four cases is shown in Fig 4, where the total expected costs are presented. Fig 4 illustrates that in case 1, which has a low probability of occurrence of any disruptive event, no investments in resilience are necessary. In fact, one can state that, when the probability of scenario {no failure} is high, the model does not suggest investments in resilience.

Moreover, in analysing Fig 4, we observe that, as the probability of scenario {no failure} decreases, the total expected cost considerably increases. In fact, comparing cases 1 and 2, the expected total cost was approximately 5 times greater in case 2 than in case 1. Additionally, compared to case 1, the total expected cost of case 4 increased drastically from $ 716,370 to $ 5,049,530. This significant increase is justified by the increases in IS, PCR and IDR values as the probability of {no failure} decreases. For case 1, the highest penalties (related to unmet demand) are observed in scenarios {LP1}, {LP2}, {LP3} and {LP4}, comprising 17% of the total expected penalty. For case 2, there was an investment of $ 1,400,000 in IDR.

**Fig 5:**
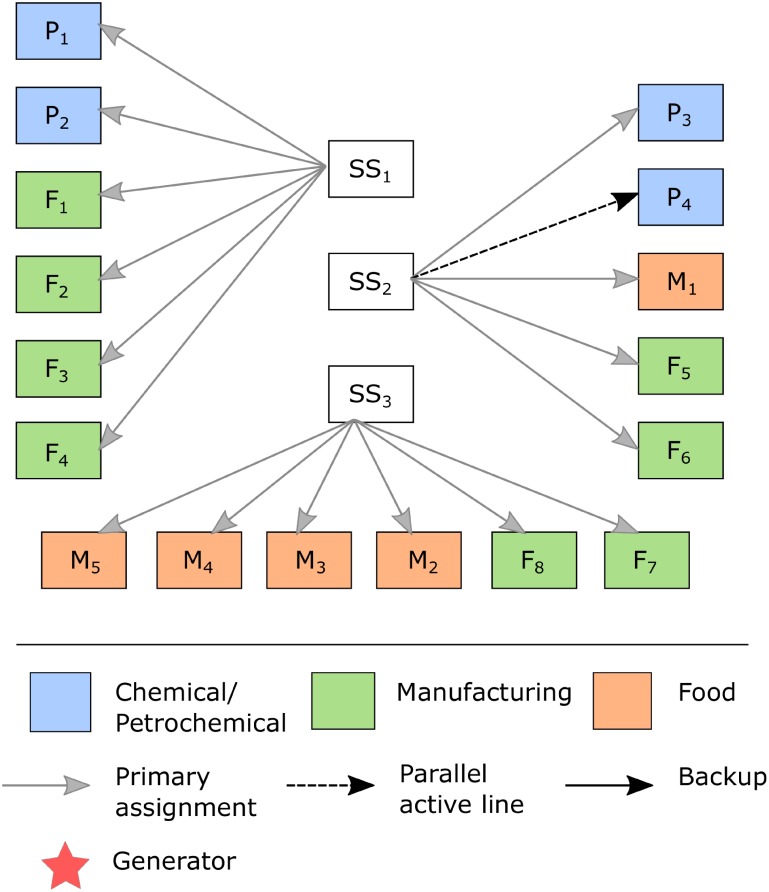
Resilience enhancement actions defined for case 3.

Fig 5 shows for case 3 that an active parallel subtransmission line (dashed line) should be added for client P_4_, which is the highest penalty related to unmet demand (chemical/petrochemical sector), as shown in Table 6. The investment in this resilience-based alternative assures that P_4_ has its demand fully met when its main subtransmission line is affected. Consequently, the penalty for the {LP_4_} scenario decreased from $86,000 in case 2 to zero in case 3. In addition, this design feature, while maintaining the operation of the system, is also used to share the workload with the main subtransmission line. It is important to note that, in practice, the design and installation of redundant lines connected to the same SS consider a distance criterion to avoid one tower falling onto an adjacent line.

**Fig 6:**
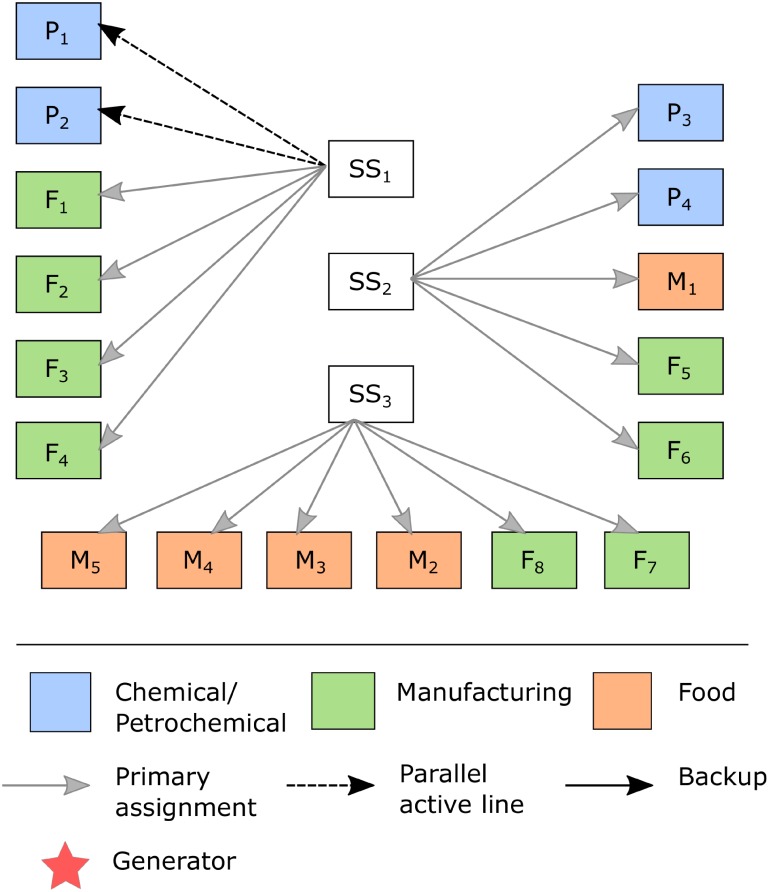
Resilience enhancement actions defined for case 4.

In case 4, the solution of the model suggested active parallel subtransmission lines for clients P_1_ and P_2_ (Fig 6). Comparing cases 3 and 4, after investing in redundant subtransmission lines, the penalty related to unmet demand for the {LP_1_} and {LP_2_} scenarios decreased from k$ 362 in case 3 to zero in case 4. As in case 2, there was also a recommendation to invest in restorative capacity for cases 3 and 4, causing an increase in the SS recovery rate of 5 MVA/hour; i.e., it increased from 20 MVA/hour to 25 MVA/hour.

Investment in active parallel subtransmission lines and in restorative capacity seems reasonable since the probability of each scenario remains low, although the probability that an event could impact a subtransmission line is considered to be ten times greater than the probability of an event that could affect an SS. However, although the cost of adding a single 2 MVA diesel generators is approximately 25% less, this action would not be as efficient as the parallel active subtransmission line in cases 3 and 4 because it would not enable the system to supply the client’s entire demand. For example, a petrochemical client would have to invest in eight generators to ensure that its demand supply was met during disruption, and the cost of this action would be approximately six times greater than that of investing in an active parallel subtransmission line.

### Assessment of the constraint on financial resources

**Fig 7:**
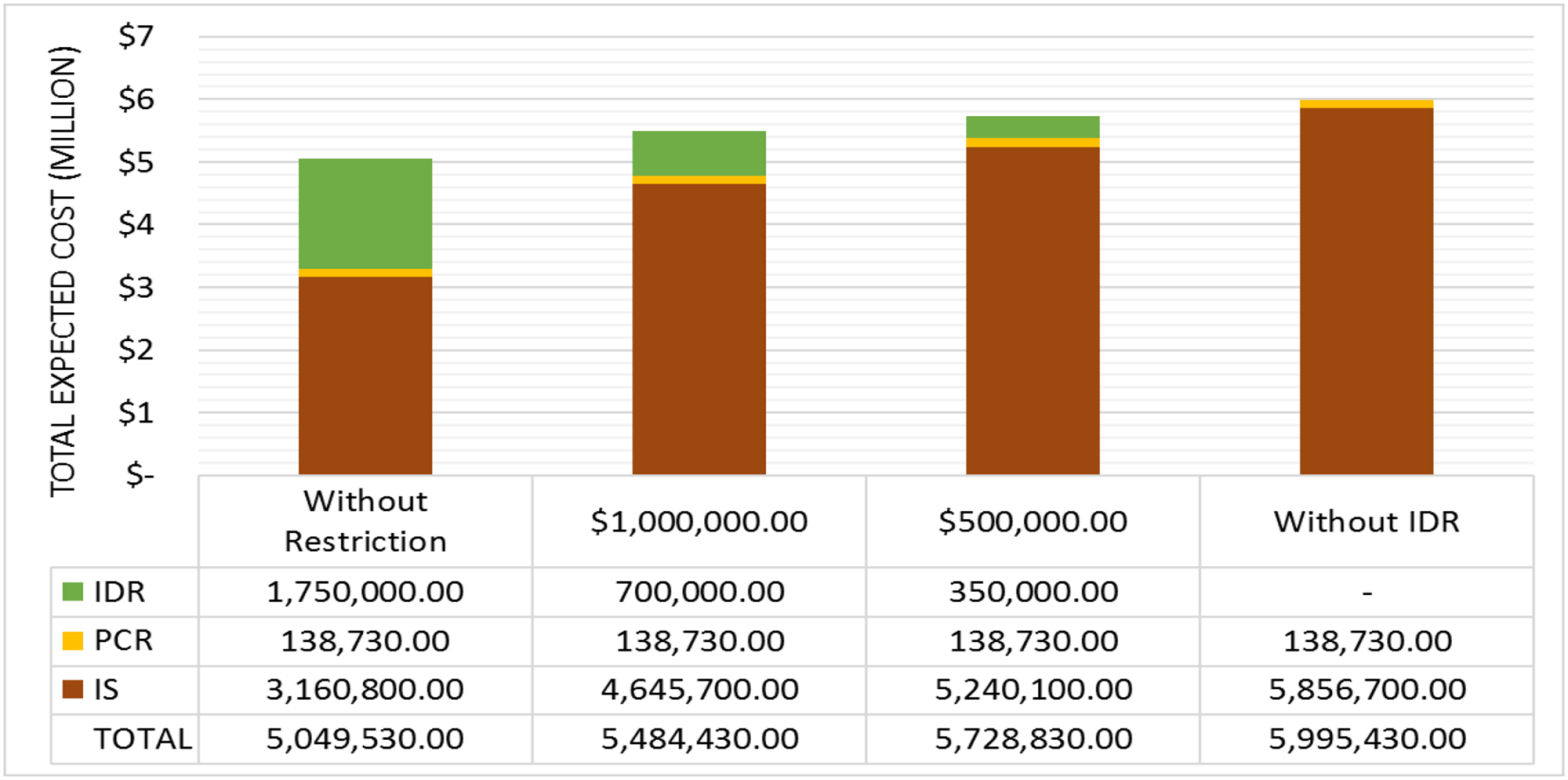
Total expected costs for case 4 for different constraints on financial resources.

In this section, we evaluate the impact of budget constraints on defining the optimal resilience-based strategy and hence on system performance. In the proposed model, the financial constraint is represented by the parameter M, which limits the investments in resilience enhancement actions (pre-event actions) and the costs associated with post-event recovery (see Constraint 26). Thus, we analyse case 4 for three different new possibilities: (i) M = $ 1 million; (ii) M = $ 0.5 million; and (iii) no investment in actions to enhance resilience (“without IDR”); the results are shown in Fig 7.

As shown in Fig 7, as M decreases, the cost associated with the impact on the system (IS) increases. For example, from the “without restriction” case to M = $ 1 million and M = $ 0.5 million, IS increases by approximately 15% and 44%, respectively. Consequently, the total expected cost also increases. Therefore, the reduction in M directly impacts the decisions on drawing up a resilience-based strategy and hence on the system performance to meet demands.

Note also that PCR does not change in the situations presented in Fig 7 because (i) all of them represent the same case 4, with all 209 scenarios and their respective likelihoods, and (ii) the system must fully recover over the time period of 8 hours (see Constraints 20 and 23). Thus, it is important to note that increasing IDR does not indicate that the PCR will be reduced because a certain total amount of resources will always be needed to perform the recovery actions associated with the disruptive event, regardless of IDR.

### Further assessments: Evaluating specific scenarios

It is also important to emphasize the flexibility that the model offers to propose solutions for a given particular event. Thus, we analyse two different scenarios to identify the optimal resilience-based strategy considering the occurrence of (i) failure of SS_1_ (scenario {S_1_}) and (ii) simultaneous failure of SS_1_SS_2_ (scenario {S_1_S_2_}). We believe that these disruptions are related to severe consequences; thus, we analyse the resilience actions that are appropriate for each of them. To this end, for each scenario, we consider its probability of occurrence equalling 1; thus, the other events in Table 4 will not occur.

#### Assessment of failure of substation SS_1_

**Fig 8:**
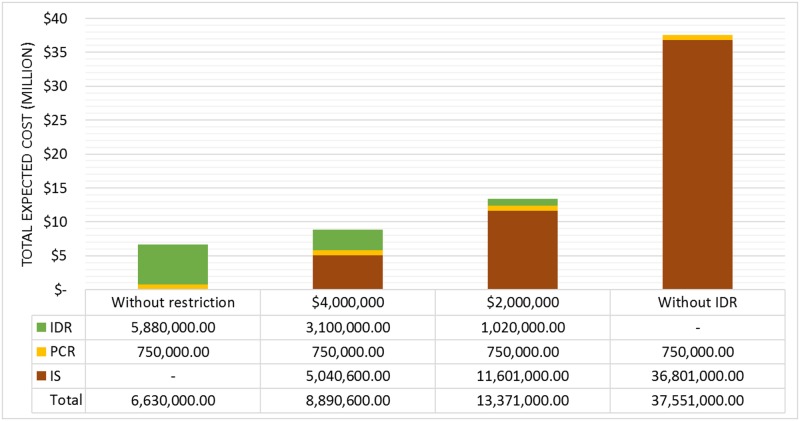
Total expected costs for scenario {S1} for different constraints on financial resources.

We evaluate this scenario for 4 investment possibilities. First, we disregard financial resource constraints (the “without restriction” case). Next, we consider M = $ 4 and M = $ 2 million. Finally, we consider the worst-case situation with no investments in resilience enhancement actions (the “without IDR” case); the results are shown in Fig 8.

As in the previous case, Fig 8 also shows that, when M is reduced, the costs associated with the system impact IS, and expected total cost increases, affecting the decisions in the elaboration of the strategy based on resilience. Thus, the expected total cost for the "without IDR" case is almost six times greater than that for the "without restriction" case.

**Fig 9:**
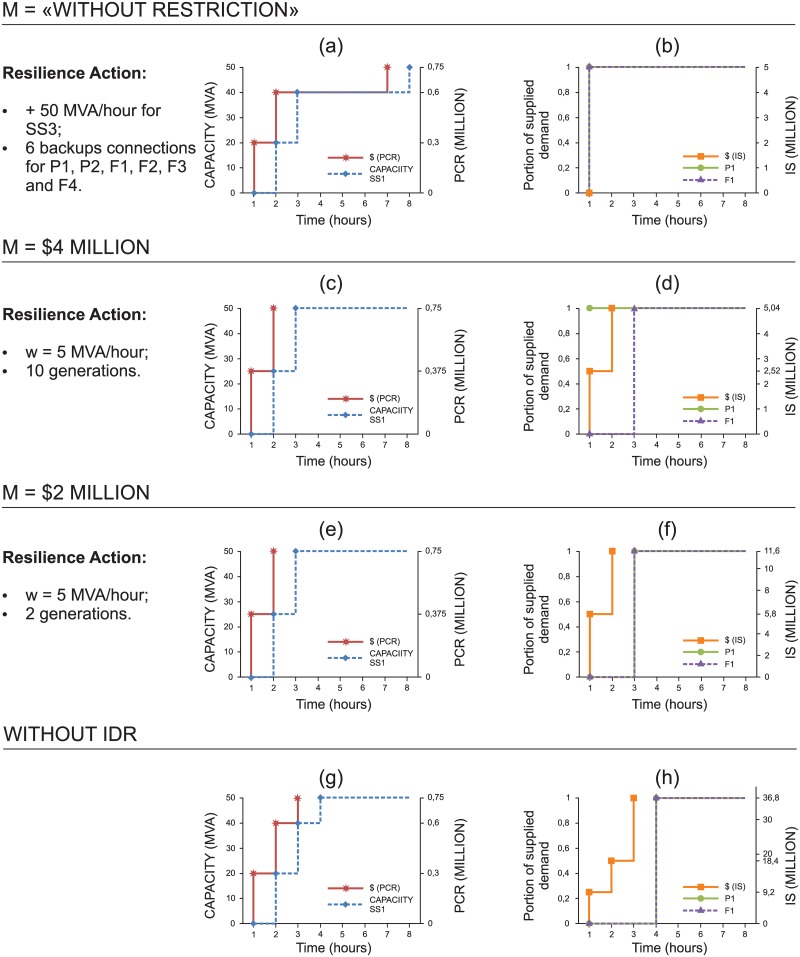
Assessment of different budgets for scenario {S1} over time. Figures (a, c, e, g) show the capacity recovery of SS_1_ and post-interruption cost recovery (PCR) for M = “Without restriction”, $ 4 and $ 2 million and “without IDR”. Figures (b, d, f, h) present the supply portion that meets the demand of customers P1 and F1 and IS for M = “Without restriction”, $ 4 and $ 2 million and “without IDR”. In addition, for each M, there is a list of resilience strategies employed on the left side of each figure.

Fig 9 shows the investments that should be made to enhance power grid resilience for each budget. These investments are assessed according to the performance of the SS recovery and the extent to which the supply of electricity meets the client’s demand, which is directly affected by the resilience actions undertaken during the downtime of the corresponding SS. We evaluate the impacts on clients P_1_ and F_1_, considering the portion of their demands supplied in scenario {S_1_}; these clients were selected to evaluate performance in supplying power to the industrial sector. The recovery speed of SS_1_ and the costs associated with PCR and IS are also illustrated in Fig 9.

According to Fig 9, higher budgets (M) emphasize investment to minimize the portion of unmet demand, while lower budgets show increased IS. In contrast to the previous cases, note that, when we consider the unavailability of SS_1_, the investments for the “without restriction” case yield improvement in the absorptive and adaptive capacities. Indeed, we can see in Fig 9 that the model suggests that (i) 6 backups connections should be established (P_1_, P_2_, F_1_, F_2_, F_3_ and F_4_) so that the clients can be supplied by SS_3_ and (ii) additional capacity should be added to SS_3_ so that it will be able to supply the additional demand.

Because SS_1_ clients would be fully supplied by SS_3_ (Fig 9b), recovery of SS_1_ would only be completed in T = 8 h (Fig 9a), as Constraint 23 requires. However, note that in Fig 9c (M = $4 million) the recovery of SS_1_ is faster (T = 3 h) than in Fig 9a because, in this case, we would have neither the additional capacity of SS_3_ nor the backup connections. We can also see in Fig 9 that, as the financial resources decrease, the investment focuses on improving restorative and adaptive capacities so that generators can be allocated to help addressing the most important clients, while SS_1_ is still in the process of recovering.

#### Assessment of the simultaneous failure of substations SS_1_ and SS_2_

**Fig 10:**
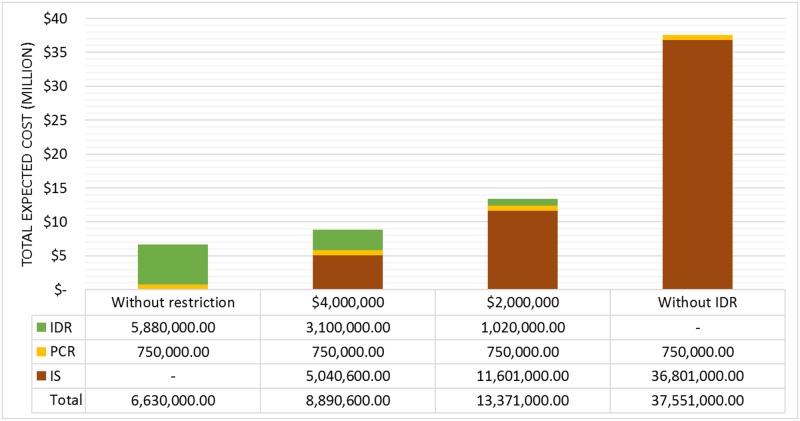
Total expected cost for scenario {S_1_S_2_} and for different financial resource constraints.

Although the probability of scenario {S_1_S_2_} is usually very low, if it occurs, it would have great impact on the performance of the system. Fig 10 shows the total cost of this event for different budget constraints. First, as in the previous section, we do not consider financial resource constraints (the “without restriction” case), and then M = $ 10, 7 and 3 million. Finally, we also consider the worst-case situation with no investments in resilience enhancement actions (the “without IDR” case).

Therefore, the optimal strategy for scenario {S_1_S_2_} has a total cost of $13,500,600: approximately 88% less than the case in which no investments in resilience are made. In fact, IS represents 16% of total expected costs for the “without restriction” case and 98% for the “without IDR” case. This finding emphasizes that investments in pre-event actions to enhance resilience (including investments in adaptive, absorptive and restorative capacities) have the potential to enable better allocation of the available financial resources to improve the efficiency of the response if disruptive events occur.

Note that, as explained for case 4, PCR remains constant for all situations presented in Fig 10 since all of them represent the occurrence of scenario {S_1_S_2_}, and the system must fully recover over the time period of 8 hours (see Constraints 20 and 23). However, PCR is much greater for scenario {S_1_S_2_} than for case 4 because we would then have more severe consequences.

**Fig 11:**
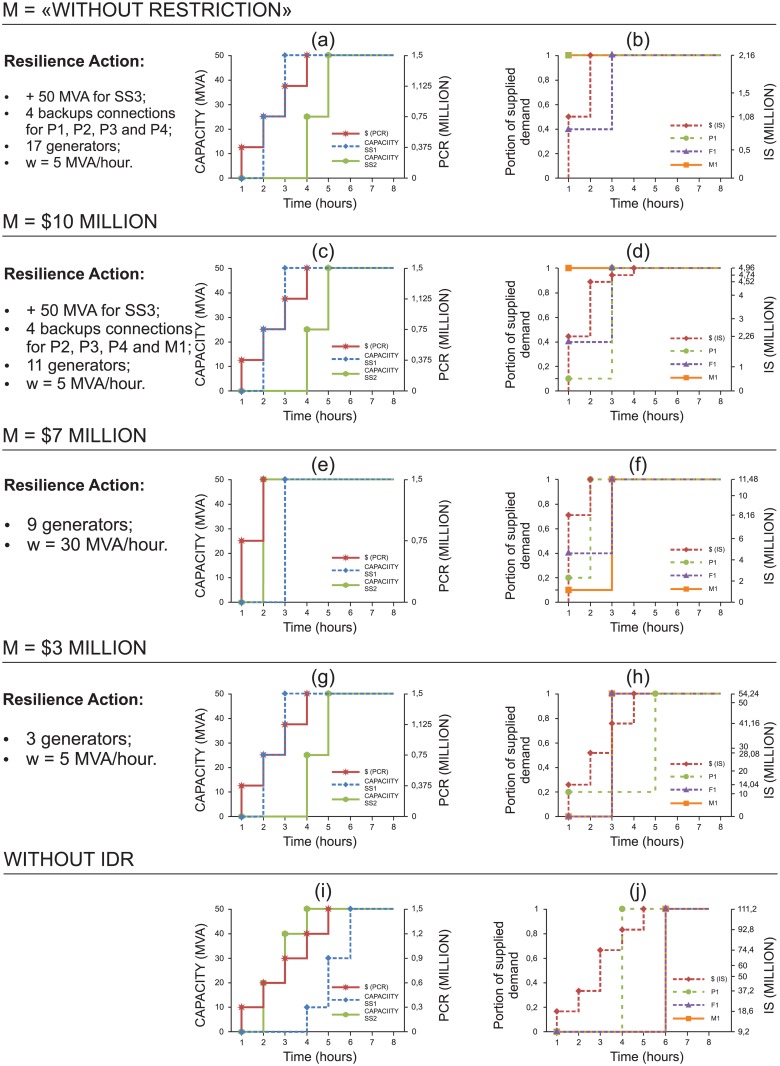
Assessment of different budgets for scenario {S_1_S_2_} over time. Figures (a, c, e, g, i) show the capacity recovery of SS_1_ and PCR for M = “without restriction”, $ 10, 7 and 3 million and “without IDR”. Figures (b, d, f, h, j) present the supply portion that meets the demand of customers P1, M1 and F1 and the cost of IS for M = “without restriction”, $ 10, 7 and 3 million and “without IDR”. In addition, for each M, there is a list of resilience strategies employed on the left side of each figure.

**Fig 12:**
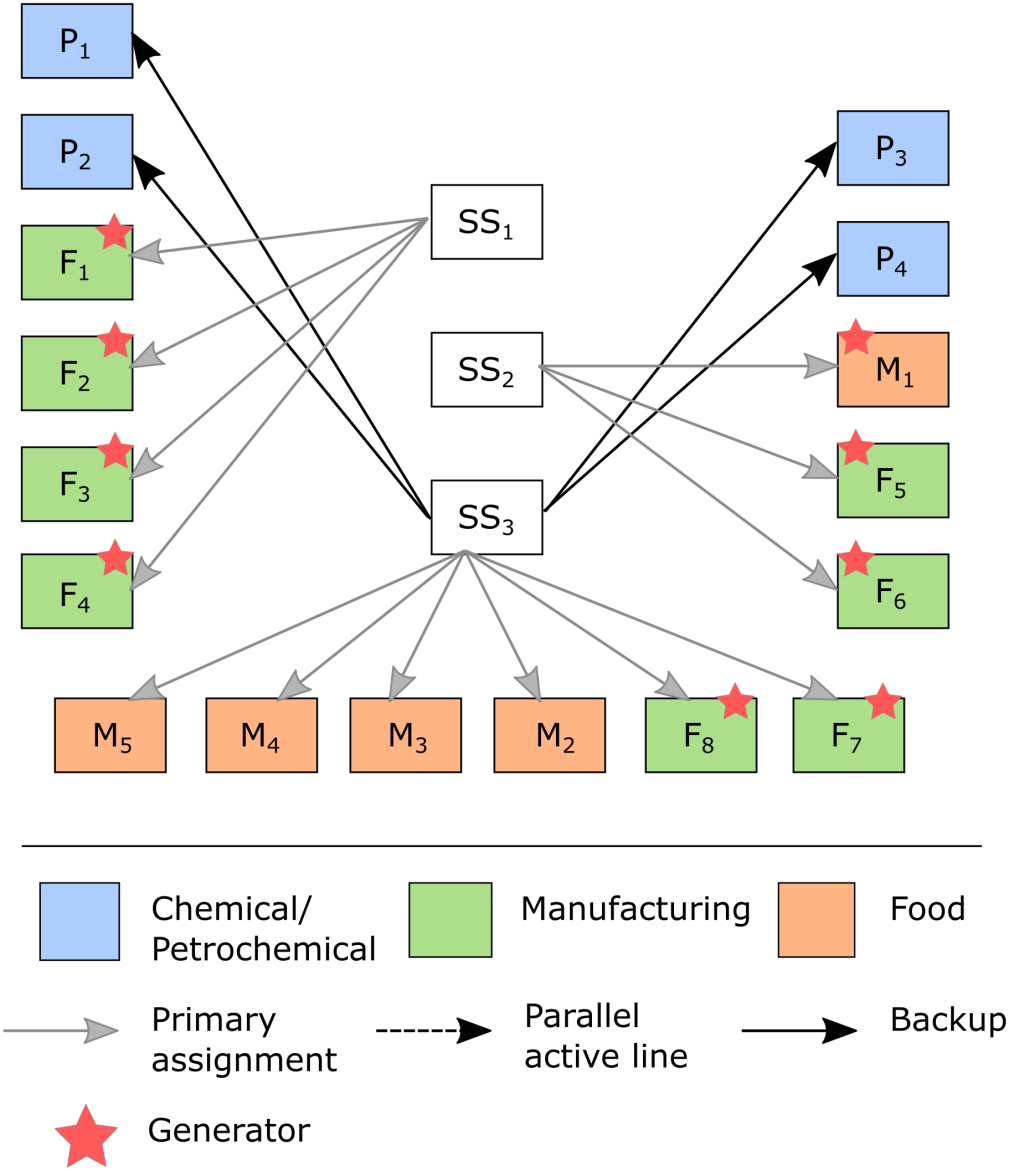
Resilience enhancement actions defined for scenario {S1S2} for M = “without restriction”.

For the “without restriction” case, according to Fig 11, the resilience actions are (i) acquiring 17 diesel generators; (ii) establishing 4 backup connections from SS_3_ to P_1_, P_2_, P_3_ and P_4_ (Fig 12); (iii) investing in additional capacity to SS_3_ (50 MVA) to accommodate the backup connections; and (iv) investing in increasing the recovery rate (w = 5 MVA/hour). Note that (i) and (ii) are related to adaptive actions, whereas (iii) and (iv) concern absorption and restoration actions, respectively.

Although the investment in the recovery rate seems small, note that each SS can only be stated as operational when at least K_j_ of its capacity (50 MVA in this case) is fully recovered. Thus, this investment allows for the recovery of SS_1_ to be completed in d = 3 hours (see Fig 11a). Although SS_1_ and SS_2_ have the same demand in MVA, note that SS_2_ has more clients, which are ranked higher in importance than SS_1_ (see Fig 3). Thus, the penalties would be higher if the clients of SS_2_ are not rapidly supplied. In this manner, the model prioritizes pre-event (adaptive and absorptive) actions to enhance resilience for SS_2_ clients, and it determines recovery strategies for SS_1_.

However, the sum of the clients’ demands would be allocated as backup to SS_3_ (P_1_, P_2_, P_3_, P_4_), exceeding its additional capacity by 10 MVA and thus indirectly affecting the supply of its own clients. In fact, clients P_1_, P_2_, P_3_, and P_4_ are prioritized because they have greater importance than the clients of SS_3_. To reduce this consequence, generators could be added to some clients of SS_3_, such as F_7_ and F_8_. In this case, after an interruption, because P_1_ is connected to SS_3_ by means of a backup connection, its demand is not affected (Fig 11b).

Table 7 shows the allocation of generators to each client; for the “without restriction” case, we also show the portion of their demand supplied by generators during SS_1_ and SS_2_ downtime. For instance, even during SS_2_ downtime, M_1_ will have 100% of its demand supplied because 5 diesel generators have been added (Fig 11b). In contrast, only 1 generator was allocated to F_1_. Because the supply capacity of the diesel generator is 2 MVA/hour, the supply of 40% of its demand is ensured until SS_1_ is fully recovered by period d = 3 (Fig 11b). Therefore, this allocation actually reduces the overall expected penalties incurred due to unmet demand. Thus, by adopting this strategy, only 5% of the total demand originally allocated to SS_3_ would not be supplied during concomitant SS_1_ and SS_2_ downtime.

**Table 7:** Allocation of diesel generators for scenario {S_1_S_2_}. Financial constraints (in millions).

Client	Without restriction		$ 10	$ 7	$ 4	$ 3	Without IDR
	No. of generators	Portion of demand supplied (%)	No. of generators				
P1	-	-	1	1	1	-	-
P2	-	-	-	1	-	-	-
F1	1	40	1	1	-	-	-
F2	1	40	1	1	-	-	-
F3	1	40	1	1	-	-	-
F4	1	40	1	1	-	-	-
P3	-	-	-	1	1	1	-
P4	-	-	-	1	1	1	-
M1	5	100	-	1	1	1	-
F5	3	100	3	-	-	-	-
F6	3	100	2	-	-	-	-
F7	1	40	1	-	-	-	-
F8	1	40	-	-	-	-	-
M2	-	-	-	-	-	-	-
M3	-	-	-	-	-	-	-
M4	-	-	-	-	-	-	-
M5	-	-	-	-	-	-	-
Total	17	-	11	9	4	3	-

For M = $10 million, the number of diesel generators was reduced by 35% (Table 7), and the 4 backup connections were now from SS_3_ to P_2_, P_3_, P_4_ and M_1_. In the “without restriction” case, the backup allocation to SS_3_ affected the supply of its own clients (F_7_ and F_8_), which no longer occurs. However, in this case, supplying the demand of P_1_ is greatly affected, as shown in Fig 11b, since only one generator is allocated to P_1_ (Table 7). For client M_1_, because it has SS_3_ by means of a backup connection, its demand is not affected. Conversely, F_1_ remains with one generator, thus ensuring the supply of 40% of its demand until SS_1_ is fully recovered. In this case, three clients of SS_2_ are also connected through backup to SS_3_ (P_3_, P_4_ and M_1_). Thus, to minimize the impact, SS_1_ should be recovered before SS_2_ (Fig 11c), and the demand of their clients (P_1_ being one of them) is supplied normally from period 3 (Fig 11d).

For M = $ 7 million, the total number of diesel generators decreases to 9, and the resilience strategy adopted for this case is more reactive because the highest amount of investment is directed to accelerating the recovery rate, which increases from 20 MVA/hour to 50 MVA/hour (w = 30 MVA/hour). Thus, the resources for SS recovery are shared between SS_1_ and SS_2_ so that both return to normal operation by the deadline d = 3 (Fig 11e). Another important point is that the fastest recovery speed was achieved for M = $ 7 million, even when compared to the case “without restriction” and M = $ 10 million.

For M = $ 3 million, investment is still made in (i) accelerating the recovery rate (w = 5 MVA/hour) and (ii) one generator each for clients P_3_, P_4_ and M_1_. The recovery speed is similar to what was presented for the “without restriction” case and M = $ 10 million, the recovery of SS_1_ being completed in three hours and that of SS_2_ in five hours (Fig 11g). However, the results for the supply meeting the demand in this case are worse than those presented for M = $ 10 million (Fig 11b). Fig 11i and 11j also illustrate the worst situations (“without IDR” case), in which no resilience enhancement actions are implemented during the design phase.

Briefly, we can note that when the budget reduces, the cheapest strategy is to invest in (i) acquiring diesel generators and (ii) accelerating recovery. As mentioned before, using generators can reduce the impact of an event on the system because doing so can keep critical, industrial equipment in minimal operating condition until the power supply returns to normal. For petrochemical clients, for example, the generators can be used to remove the work in process and to allow the system to restart without any further delays when the power supply returns.

**Fig 13:**
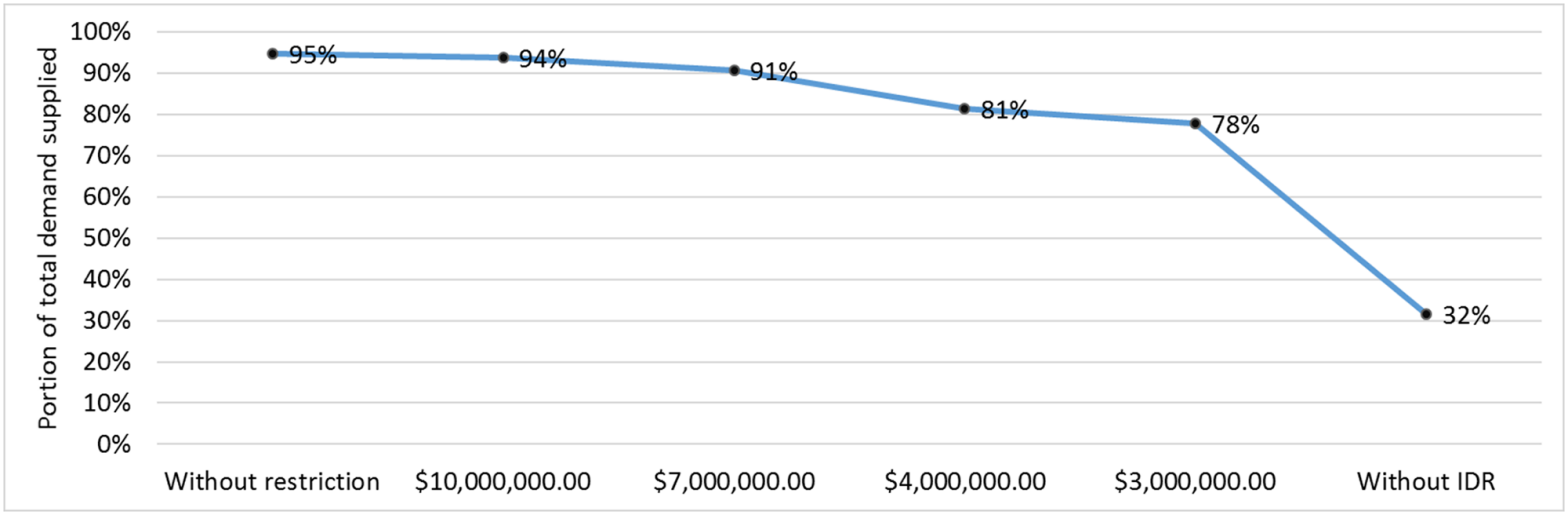
Assessment of the total demand for supply met over the period of 8 hours for different financial resource constraints.

However, Fig 13 illustrates the portion of the overall demand supplied in each situation, considering the performance for all clients over the 8-hour period. Fig 13 indicates that actions towards incorporating the absorption and adaptation capacities enable the response to be more effective than actions that focus on recovery. Moreover, our model reflects that it is economically unfeasible to ensure that 100% of the demand will be met should disruptive events occur. However, we can minimize the impact on the system (IS) by adopting pre-event resilient actions.

## Conclusions

This paper proposed a model to optimize costs in the design phase of an EPSN related to industrial clients when resilience-based actions are considered. The MILP model developed was able to incorporate (i) several disruptions with their respective probabilities of occurrence and (ii) worst-case scenarios, in which a specific event with severe consequences is considered. In the first situation, the probabilities of occurrence of each of the mutually exclusive scenarios are considered, and the output of the model is the optimal strategy involving pre- and post-event actions that minimize the expected total cost. We assessed four different cases by varying the probability of the event {no failure}. In the second situation, the probability of the selected scenario was set to 1, while the probabilities of all other scenarios were 0. We evaluated the scenarios {S_1_} and {S_1_S_2_} involving the loss of SS_1_ and the joint failure of SS_1_ and SS_2_.

The model was validated by two types of sensitivity analysis. First, we increased the probability of the occurrence of an undesired event. From the results, we can see that our model indicated that the decision maker should also increase investments to design a more resilient system. In contrast, by reducing the probability of occurrence, no investment should be made. Thereafter, we also evaluated how the model behaves for different budgets. As expected, as we decreased the budget, the IS increased rapidly, indicating the usefulness of investing in resilience during the design phase. Note that the proposed model also indicated how the resources should be spent for each case.

The results obtained enabled the optimal solution to be analysed in terms of IS, IDR and PCR. Moreover, detailed IDR actions (e.g., redundant or backup lines, diesel generators) are real-world suggestions to improve the resilience of EPSN related to industrial clients. Thus, the impacts on EPSN clients due to disruptions were reduced, as evidenced in the sensitivity analysis, in which IS increased by reducing the investments in resilience strategies. This analysis also showed that the lower the investment in IDR, the greater the level of unmet demand, which can yield financial losses for the entire system.

Another important contribution is to draw attention to a paradigm change in how a power grid is viewed: the traditional stance is that the grid is system centred on electric power utilities. However, the new paradigm is that the grid is not only system centred but is also a customer-focused system, which is the reasoning followed by other authors, such as Kwasinski [64]. Therefore, our model includes strategies that can be applied both to electric power grids and by industrial customers. For example, such strategies include considering redundant or backup systems and diesel generators, thus allowing customers to make decisions about managing electric power, which has a strong influence on enhancing the overall resilience of the entire grid.

We point out some limitations of this work. First, we focused on adopting the “resilience triangle” concept. However, other capacities or strategies for resilience do exist and they can be the focus of future research. For example, Lundberg [72] suggests considering the “learning” capacity to monitor and anticipate a disaster. Another possibility is to deem structural changes to increase the absorptive capacity of the system against shock (e.g., Raby et al. [73]). Moreover, we have considered the objective function as a weighted average of the costs of a set of possible interruption events, each with its respective probability. This could be thought of a limitation because, for example, low-probability high-consequence and high-probability low-consequence events are considered similar for resource allocation purposes. Despite that, the model allowed us to investigate specifically high-consequence events such as the failure of SS_1_ and the simultaneous loss of SS_1_ and SS_2_.

Finally, developing a multi-objective optimization model is an issue of our ongoing research. In fact, we aim at minimizing the total costs related to the three resilience capacities (absorption, adaptation and recovery), as well as maximizing the level of service to industrial customers. Other topics of ongoing research involve (i) analysing how local energy storage can contribute to rendering the electric service at an industrial plant more resilient to disruptions and (ii) for more fine-grained networks, although the proposed MILP is still valid, investigating a method that uses a metaheuristic solution (e.g., genetic algorithms) is an alternative due to the greater number of system nodes and links.

## Abbreviations

CI: Critical Infrastructure
EPSN: Electric Power Supply Network
IDR: Investments in Design for Resilience
IS: Impact on System
KPG: South Korean Power Grid
MILP: Mixed-Integer Linear Programming
PCR: Post-interruption Cost of Recovery
SS: Subtransmission Substations
TS: Transmission Substations
